# Exposure Without Active Infection: Surveillance of Influenza A Viruses and Coronaviruses in Antarctic Seabirds

**DOI:** 10.3390/v18020248

**Published:** 2026-02-15

**Authors:** Jennifer Oliveira Melo, Leonardo Corrêa da Silva Junior, Martha Lima Brandão, Bruno Rocha Pribul, Luciana Trilles, Roberto do Val Vilela, Dilmara Reischak, Marilda M. Siqueira, Paola Cristina Resende, Maria Ogrzewalska

**Affiliations:** 1 Laboratory of Respiratory Viruses, Exanthematic, Enteroviruses and Viral Emergencies, Oswaldo Cruz Institute, Fiocruz Foundation, Avenida Brasil 4036, Manguinhos, Rio de Janeiro 21041-361, RJ, Brazil; leocorrra15@gmail.com (L.C.d.S.J.); marildamts@gmail.com (M.M.S.); paolabmrj@gmail.com (P.C.R.); 2 Production and Innovation in Health, Fiocruz Foundation, Avenida Brasil 4036, Manguinhos, Rio de Janeiro 21041-361, RJ, Brazil; brandaomarthal@gmail.com; 3 Laboratory of Bacteriology Applied to One Health and Antimicrobial Resistance, Fiocruz Foundation, Avenida Brasil 4036, Manguinhos, Rio de Janeiro 21041-361, RJ, Brazil; bruno.pribul@ioc.fiocruz.br; 4 Mycology Laboratory, National Institute of Infectious Diseases, Evandro Chagas, Fiocruz Foundation, Avenida Brasil 4036, Manguinhos, Rio de Janeiro 21041-361, RJ, Brazil; luciana.trilles@fiocruz.br; 5 Laboratory of Biology and Parasitology of Reservoir Wild Mammals, Oswaldo Cruz Institute, Fiocruz Foundation, Avenida Brasil 4036, Manguinhos, Rio de Janeiro 21041-361, RJ, Brazil; roberto.vilela@fiocruz.br; 6 Federal Agricultural Defense Laboratory, Ministry of Agriculture and Livestock, R. Raul Ferrari, Jardim Santa Marcelina, Campinas 13100-105, SP, Brazil; dilmara.reischak@agro.gov.br

**Keywords:** Antarctic seabirds, penguins, influenza A virus, serology, viral surveillance

## Abstract

Understanding the circulation of influenza A viruses and other respiratory pathogens in Antarctic wildlife is essential for anticipating outbreaks and evaluating potential impacts on vulnerable populations. During the austral summer of December 2024 and January 2025, we conducted viral surveillance in six bird species breeding at Lions Rump, King George Island, South Shetland Islands, Antarctica. A total of 199 individuals were sampled, including *Pygoscelis papua* (gentoo penguin; *n* = 81), *Pygoscelis adeliae* (Adélie penguin; *n* = 79), *Pygoscelis antarcticus* (chinstrap penguin; *n* = 34), *Stercorarius antarcticus* (brown skua; *n* = 2), *Chionis albus* (snowy sheathbill; *n* = 2), and *Eudyptes chrysolophus* (macaroni penguin; *n* = 1). All cloacal and oropharyngeal swabs tested negative for influenza A viruses and coronaviruses by RT-PCR. Blood samples from 177 birds were screened by enzyme-linked immunosorbent assay, which detected influenza A virus antibodies in 20 individuals (11.3%). Hemagglutination inhibition assays identified subtypes H6 and H11 in two penguins and H1, H5, H6, and H9 in one skua. These findings reveal no evidence of active viral infection during the sampling period but provide serological evidence of past exposure in seabird populations at Lions Rump. Continued surveillance is essential to characterize viral dynamics in Antarctic ecosystems and to support early detection and preparedness for potential incursions of emerging high-pathogenicity influenza A viruses.

## 1. Introduction

The worldwide circulation of the high-pathogenicity avian influenza (HPAI) H5N1 virus clade 2.3.4.4b has led to exceptional levels of mortality in wild species [1]. By the end of 2022, B3.2 genotype viruses reached the Pacific coast of South America, spreading quickly and triggering outbreaks in both domestic birds and wildlife [2,3,4]. During October and November 2023, HPAI H5N1 expanded from South America into the Subantarctic region, leading to outbreaks among seabirds and marine mammals in South Georgia and the Falkland (Malvinas) Islands [5,6,7]. As HPAI H5N1 continued to circulate among wildlife in South America and South Georgia, concerns grew that the virus might advance into the Antarctic Peninsula [8,9]. The first reports of wildlife deaths suspected to be caused by H5N1 in the Antarctic region emerged in December 2023 [10], and multiple confirmed detections have since been documented throughout the Antarctic Peninsula [10,11,12,13].

The presence of influenza A viruses in Antarctica has been documented for more than five decades. Early studies reported antibodies in brown skuas *Stercorarius antarcticus* Lesson, 1831; Adélie penguins *Pygoscelis adeliae* Hombron & Jacquinot, 1841; chinstrap penguins *Pygoscelis antarcticus* (Forster, 1781) and gentoo penguins *Pygoscelis papua* (Forster, 1781), among other species, with most investigations relying primarily on serological assays to infer previous exposure [14,15,16,17,18,19,20]. Subsequent research incorporated molecular detection and viral sequencing, confirming the persistent and diverse circulation of various low-pathogenicity avian influenza viruses (LPAIV), including strains of the subtypes H11N2, H5N5, H6N8, and H4N7, as well as H1N1 and H3N8, in multiple species [18,19,21,22,23,24].

Although influenza A viruses have been investigated in Antarctica for several decades, most studies have focused on either serological evidence of past exposure or molecular detection of viral RNA, but rarely both within the same sampling framework. This separation has limited the ability to determine whether viral detections reflect active circulation, residual exposure from earlier introduction events, or broader patterns of viral persistence in Antarctic wildlife. In the context of the recent introduction of high-pathogenicity avian influenza H5N1 into the region, addressing this methodological gap is particularly important to accurately interpret infection dynamics, assess viral persistence and re-introduction risk, and better understand ecological connectivity between the South American continent and the Antarctic Peninsula.

In addition to influenza, surveillance efforts have expanded to include avian coronaviruses (CoVs), whose diversity and evolutionary plasticity have attracted increasing scientific attention [25,26]. Deltacoronaviruses have recently been detected in several Antarctic bird species, such as gulls, penguins, and sheathbills [27,28,29]. Their presence underscores the importance of continued monitoring, as environmental and climatic changes may facilitate the emergence of variants capable of adapting to new hosts posing a potential risk to ecological and human health [27,30].

There is still limited knowledge about active viral circulation in live Antarctic birds during the early stages of the current H5N1 incursion. The contribution of endemic LPAIs and their potential interactions with the newly introduced H5N1 strain also remain unknown. Monitoring healthy, free-ranging birds is therefore essential, as it can reveal subclinical infections, recent introduction events, historical exposure to multiple subtypes, and potential differences between penguin species and predatory or scavenging birds. In this context, the present study investigated the presence of influenza A viruses and avian coronaviruses in marine birds sampled on King George Island, South Shetland Islands, during the 2024/2025 austral summer.

## 2. Materials and Methods

**Figure 1:**
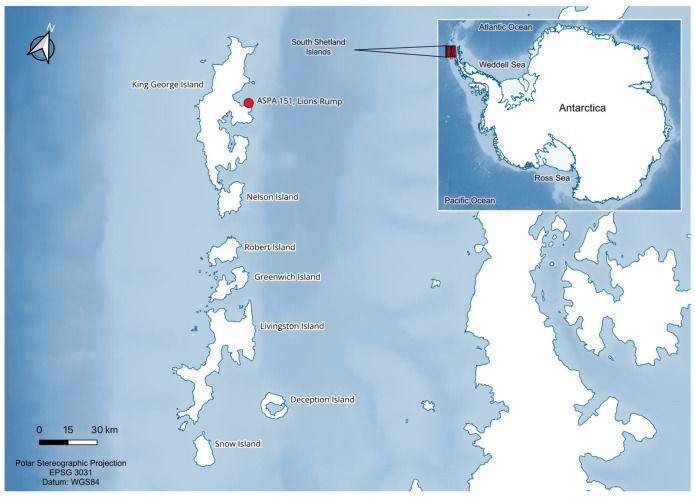
Map of the South Shetland Islands showing the location of Antarctic Specially Protected Area (ASPA) 151 in Lions Rump, King George Island (red dot). Map produced in QGIS using datasets from Quantarctica, Norwegian Polar Institute [32].

The birds were captured with hand nets in a breeding colony within Antarctic Specially Protected Area ASPA 151 (62°08′ S; 58°07′ W), Lions Rump, King George Island, South Shetland Islands (Figure 1), during the austral summer of December 2024–January 2025. Lions Rump occupies 1.61 km^2^ on the southwestern coast of King George Island and is an important breeding site for several seabird species, including Adélie penguins, chinstrap penguins and gentoo penguins. The area is characterized by high seabird density and species diversity, as documented in previous monitoring efforts. In the 2023/24 season, 2716 Adélie, 3769 gentoo, and 39 chinstrap penguin nests were recorded [31]. Eight additional bird species also breed in the area, including cape petrels *Daption capense* (Linnaeus, 1758); Wilson’s storm petrels *Oceanites oceanicus* Kuhl, 1820; black-bellied storm petrels *Fregetta tropica* (Gould, 1844); snowy sheathbills *Chionis albus* Gmelin, 1789; south polar skuas *Stercorarius maccormicki* Saunders, 1893; brown skuas *Stercorarius antarcticus*; kelp gulls *Larus dominicanus* Lichtenstein, 1823; and Antarctic terns *Sterna vittata* Gmelin, 1789. The beaches also host substantial numbers of southern elephant seals *Mirounga leonina* (Linnaeus, 1758) and Antarctic fur seals *Arctocephalus gazela* (Peters, 1875). Human disturbance has been very limited, apart from sporadic monitoring programs focused on bird and mammal populations and some geological or geomorphological research [31].

Penguins were captured using hand-held mist nets while moving from the breeding colony toward the sea, in order to avoid disturbing individuals on their nests. Sheathbills and Skuas were captured opportunistically. Sheathbills were captured near their nests in rock cavities, and skuas were caught either on nests or while scavenging penguin chick carcasses. This approach minimized disturbance while allowing safe handling and sampling. Each bird was visually inspected for general condition, behavior, and physical abnormalities, but no clinical symptoms were observed in any of the individuals.

From each individual, one cloacal swab, one oropharyngeal swab, and a blood sample were collected. Cloacal and oropharyngeal swabs were obtained using sterile swabs and immediately placed into 1 mL of RNA/DNA Shield (Zymo Research, CA, USA) to preserve nucleic acids. Blood samples were collected using sterile syringes and transferred directly into sterile Eppendorf tubes. In penguins, blood was obtained from the metatarsal vein, with a maximum volume of 4 mL collected using a 5 mL syringe fitted with a sterile 0.70 × 25 mm hypodermic needle. In skuas and snowy sheathbills, blood was collected from the tarsal vein, with up to 1 mL obtained using a 3 mL syringe fitted with a sterile 0.55 × 20 mm hypodermic needle. All procedures were conducted in accordance with ethical approval from the Animal Use Ethics Committee of the Oswaldo Cruz Foundation (CEUA-Fiocruz), protocol nº P-9/2024-3. Following collection, all samples were stored in refrigerated cooler boxes in the field for no longer than 4–5 h. Upon arrival at the field laboratory, blood samples were centrifuged, and serum aliquots were stored at −20 °C. Swab samples were likewise frozen immediately upon arrival to maintain temperature stability and preserve sample integrity. This protocol minimized the time between collection and preservation, reducing potential temperature-related effects on downstream analyses.

We strictly followed protocols developed by Brazilian Ministry of the Environment and the Brazilian Antarctic Program (PROANTAR) with the Fiocruz Biosafety Working Group. All field researchers wore Tyvek suits, double nitrile gloves for sampling, heavy-duty rubber gloves for restraining birds, N95 masks, and protective goggles. Outer nitrile gloves were changed between birds, and rubber gloves were disinfected after each handling with a water and Herbalvet solution (Benzalkonium chloride—15 g/100 mL). Feces or blood were immediately cleaned with the same solution, and sample tubes were disinfected before transport. In the field lab, protective clothing, gloves, and masks were used during sample processing. All materials were cleaned before disposal, and boots were disinfected after returning from the field. These measures prevented cross-contamination and minimized zoonotic risk among different host species. The project was conducted under authorized permits for access and sampling within ASPA, issued by the PROANTAR, and was approved by the institutional Animal Ethics Committee (protocol P-9/24-3).

In the laboratory, 140 µL of each sample was used for viral RNA extraction. RNA was extracted with the QIAamp Viral RNA Mini Kit (Qiagen, Hilden, Germany) according to the manufacturer’s instructions. The resulting RNA extracts were immediately stored at −80 °C until further molecular analyses. Negative controls, consisting of RNase/DNase-free water, together with positive controls provided by the kit manufacturer, were used to validate assay performance. Screening for influenza A virus (IAV) was performed using a one-step real-time RT-PCR assay targeting the matrix (M) gene, employing a commercial RT-PCR kit (Biomanguinhos, Rio de Janeiro, Brazil). The assay follows a previously published and validated protocol, in which primer and probe sequences as well as thermal cycling conditions are described in detail [33]. Samples exhibiting a characteristic sigmoid amplification curve with a cycle threshold (Ct) value < 38 were considered positive.

All samples were additionally screened using a pan-coronavirus PCR assay targeting the RNA-dependent RNA polymerase (*RdRp*) gene, following previously published protocols [29,34]. Briefly, RNA extracts were first subjected to a primary RT-PCR using the primers RdRp S1 (5′-GGKTGGGAYTAYCCKAARTG-3′) and RdRp R1 (5′-TGYTGTSWRCARAAYTCRTG-3′) with the One-Step RT-PCR Enzyme Mix Kit (Qiagen, Hilden, Germany), generating an expected product of approximately 602 bp. A nested PCR was then carried out with the Phusion RT-PCR Enzyme Mix Kit (Sigma-Aldrich, St. Louis, MO, USA), using the primers Bat1F (5′-GGTTGGGACTATCCTAAGTGTGA-3′) and Bat1R (5′-CCATCATCAGATAGAATCATCAT-3′) and 1 µL of the first-round amplicon as template. The secondary amplification yielded ~440-bp products, which were visualized on 1.5% agarose gels stained with SYBR Safe DNA Gel Stain (Thermo Fisher Scientific, Waltham, MA, USA).

Serum samples were screened for influenza A antibodies using a competitive enzyme-linked immunosorbent assay (cELISA) targeting antibodies against the nucleoprotein of influenza A virus in multiple species (ID Screen^®^ Influenza A Antibody Competition Multi-species, IDvet, Innovative Diagnostics, Grabels, France; FLUACA-2P). Samples that tested positive were subsequently subjected to hemagglutination inhibition (HI) assays for subtype determination against all influenza A hemagglutinin subtypes previously reported in Antarctica (H1-H7, H9-H11, H14-H15).

Prior to HI testing, sera were treated with a 10% suspension of chicken erythrocytes to remove non-specific agglutinins. The HI assay was performed in V-bottom 96-well microtiter plates using 1% chicken erythrocytes and four hemagglutinating units (HAU) of each inactivated antigen, following the methodology recommended in the World Organisation for Animal Health (WOAH) Terrestrial Manual [35]. Sera were tested in two-fold serial dilutions, and samples were considered HI-positive when complete inhibition of hemagglutination was observed at a serum dilution of ≥1:16 against 4 HAU of the respective antigen. Antigen controls, subtype-specific positive control sera, and erythrocyte controls were included in each assay. All antigens were provided by WOAH reference laboratories for avian influenza.

## 3. Results

During the austral summer expedition in December 2024–January 2025, biological samples were collected from 199 birds, comprising gentoo penguins (*n* = 81), Adélie penguins (*n* = 79), chinstrap penguins (*n* = 34), brown skuas (*n* = 2), snowy sheathbills (*n* = 2), and macaroni penguin *Eudyptes chrysolophus* (Brandt, 1837) (*n* = 1). Cloacal and oropharyngeal swabs were tested by RT-PCR. All birds had both cloacal and oropharyngeal swabs analyzed, with the exception of one gentoo penguin (*P. papua*), for which only a cloacal swab was available. All tested swab samples were negative for influenza A virus and coronaviruses.

Blood samples were obtained from 177 individuals and tested for antibodies against influenza A virus, revealing seropositivity in 20 birds (11.3%), including: 6 of 70 Adélie penguins (8.6%), 10 of 31 chinstrap penguins (32.5%), 2 of 2 brown skuas (100%), and 2 of 2 snowy sheathbills (100%). All sera from gentoo penguins (*n* = 71) and the macaroni penguin (*n* = 1) were negative. Hemagglutination inhibition assays allowed subtype characterization in two seropositive Adélie and chinstrap penguins, showing reactivity to influenza A virus subtypes H6 and H11, and in one brown skua, which showed antibodies against H1, H5, H6, and H9, with subtype-specific HI titers ranging from 1:16 to ≥1:32 (Table 1).

## 4. Discussion

Understanding the ecology of influenza A viruses (IAVs) and coronaviruses (CoVs) in Antarctica is crucial for elucidating their global movement and assessing the risks associated with the introduction and circulation of high-pathogenicity avian influenza (HPAI) viruses. Following the emergence of HPAI H5N1 in Antarctica during the 2023/2024 austral season, multiple surveillance efforts have documented viral circulation in the Antarctic Peninsula and South Shetland Islands across consecutive seasons [5,9,11,12,13,24,36,37,38,39,40].

In the present surveillance study, conducted during the 2024/2025 austral summer at Lions Rump (King George Island), no active IAV or coronavirus infection was detected by RT-PCR in cloacal and oropharyngeal swabs collected from live birds. Nevertheless, the detection of influenza A antibodies in 11.3% of sampled individuals by ELISA indicates previous exposure to IAV. Among these seropositive birds, only three individuals (15%) yielded subtype identification by hemagglutination inhibition.

These findings should be interpreted in light of both contemporaneous and earlier surveillance studies conducted in the South Shetland Islands and Antarctic Peninsula. Recent investigations at Fildes Peninsula, located on the opposite side of King George Island, documented the detection of high-pathogenicity avian influenza (HPAI) H5N1 in seabird and marine mammal carcasses, as well as in fecal samples from live skuas, during the 2023/2024 and 2024/2025 austral seasons [24,39]. In addition, our research group reported HPAI H5N1 detection in seabird and marine mammal carcasses from other sites within the South Shetland Islands during the same sampling period [37,40].

In contrast, previous studies conducted prior to the emergence of HPAI H5N1 in Antarctica reported molecular detection of influenza A virus in live Antarctic birds, including work by our research group and others [18,19,20,23], highlighting that active IAV infection has been detected in this region under different ecological and epidemiological contexts.

Several non-mutually exclusive hypotheses may help explain this apparent discrepancy. First, IAV circulation in Antarctic ecosystems appears to be spatially heterogeneous, with localized clusters of active infection that may not coincide with the specific colony sampled at a given time. Second, the host community sampled in this study was strongly dominated by penguins, whereas predatory and scavenging birds, such as skuas and sheathbills, which showed a high proportion of seropositivity despite limited sample size, were underrepresented. This sampling imbalance may have reduced the likelihood of detecting active infection, given the ecological role of these non-penguin species in virus exposure and transmission. Finally, differences in sampling strategies, particularly the focus on live, apparently healthy birds rather than carcasses or clinically affected individuals, may further contribute to variation in detection outcomes. Together, these factors underscore the importance of interpreting molecular results within the broader ecological, spatial, and methodological context of Antarctic IAV surveillance.

When interpreting our serological results, a low correspondence between ELISA positivity and successful hemagglutination inhibition (HI) subtype identification is expected because the two assays target different viral antigens and differ substantially in analytical sensitivity. The ELISA used in this study detects antibodies against the highly conserved nucleoprotein (NP), which provides broad reactivity across influenza A viruses and is widely used as a sensitive screening tool.

In contrast, the HI assay detects subtype-specific antibodies directed against hemagglutinin (HA) and requires sufficiently high antibody titers as well as a close antigen–antibody match to yield detectable inhibition [41,42]. As a result, HI is inherently less sensitive than ELISA and may fail to identify HA subtypes when antibody levels are low, even in previously exposed individuals [18,43]. In addition, the reference antigens used in our HI panel, which are derived primarily from European and North American viral isolates, may differ antigenically from hemagglutinins circulating in Antarctic birds, further reducing assay reactivity, a limitation previously reported in wildlife influenza surveillance [18]. Together, these methodological and immunological factors explain why only a small subset of ELISA-reactive sera yielded subtype identification by HI and indicate that the absence of HI reactivity does not contradict prior exposure to influenza A virus in the sampled birds.

The serological results obtained for *Pygoscelis* penguins in this study are broadly consistent with previous investigations conducted in the Antarctic region. Earlier serological surveys have generally reported low to moderate levels of influenza A virus exposure in penguins, with seroprevalence values ranging from 0 to 31% [15,16,18,20,21,44,45], comparable to the seroprevalence observed here (11.3%).

In contrast, molecular detection of active infection in penguins has been reported inconsistently across studies and appears to be highly variable in space and time. While many surveys, including the present study, failed to detect viral RNA in sampled individuals, relatively high IAV prevalence has been documented in specific colonies, such as Rada Covadonga and Aitcho Island [18,19]. These findings indicate that IAV circulation in penguins may occur in spatially and temporally restricted clusters, rather than as sustained, widespread transmission. Accordingly, one plausible interpretation of our results is that sampling at Lions Rump did not coincide with a colony experiencing an active localized outbreak at the time of the study.

Within the genus Pygoscelis, seroreactivity in our dataset was restricted to *P. adeliae* and *P. antarcticus*, whereas no antibodies were detected in *P. papua*, suggesting potential interspecific differences in exposure or susceptibility. The detection of antibodies against subtypes H6 and H11 is consistent with previous reports from Antarctic penguins, in which these subtypes, as well as H1, H3, H7, H9, and H10, have been identified by serology or sequencing [14,16,18,19,20,21,23,24,44,45,46].

On the other hand, the absence of detectable antibodies against H5 in penguins suggests no evidence of prior exposure of adult *Pygoscelis* penguins to HPAI H5N1 at the time of sampling. Although penguins share breeding areas with several scavenging and predatory species, multiple ecological factors may reduce their probability of encountering H5N1 compared with skuas and sheathbills. *Pygoscelis* penguins are primarily pelagic foragers that spend most of their time at sea and interact mainly with conspecifics, with limited contact with carcasses or with species known to amplify or disperse influenza A viruses [47,48,49]. In contrast, scavengers such as brown skuas and snowy sheathbills routinely feed on carcasses, prey on weakened birds, and frequently move between colonies, increasing their likelihood of encountering infectious material [50,51].

During recent HPAI H5N1 outbreaks in South Georgia and the Falkland Islands, mortality patterns varied among penguin species, with gentoo penguins experiencing particularly severe impacts in some regions, while scavenging birds and marine mammals were consistently among the most affected taxa [5,12,13,36,37,40]. These differences underscore the importance of species-specific ecology and exposure pathways in shaping infection risk and disease outcomes.

Among the non-penguin species sampled, both snowy sheathbills (*Chionis albus*) tested positive by ELISA, indicating that 100% (2/2) of the sampled individuals had serological evidence of prior exposure to influenza A virus. Although none of the sera reacted with the panel of tested hemagglutinin antigens, this may reflect exposure to influenza A subtypes not included in the HI panel or antigenic mismatch, rather than absence of subtype-specific antibodies. Notably, subtype H11N2 has previously been detected in *C. albus* in Antarctica [21]. Snowy sheathbills are opportunistic scavengers that maintain close contact with penguin colonies, frequently feeding on carrion, animal feces, and preying on chicks, behaviors that increase their likelihood of exposure to influenza A viruses. In addition, some individuals migrate northward to the Falkland Islands and the southernmost coastal regions of South America [52], potentially facilitating viral connectivity between South America and the Antarctic Peninsula.

Similarly, 50% (1/2) of the sampled brown skuas showed serological evidence of exposure to multiple influenza A virus subtypes, including H1, H5, H6, and H9. Exposure to influenza A viruses in skuas has been consistently reported in previous studies [17,20], and recent detections of HPAI H5N1 in skuas in Antarctica further emphasize their epidemiological relevance [5,12,13]. Skuas are highly vulnerable to H5N1 infection, a pattern reflected in documented mortality events and in their scavenging ecology, which promotes frequent contact with infected carcasses [5,12,13,36,53].

Although sample sizes for non-penguin species were small, the high proportion of seropositive individuals among sheathbills and skuas is consistent with previous work identifying these wide-ranging scavengers as particularly informative targets for influenza A virus surveillance in Antarctica [17,20,21,39,53]. These findings reinforce the value of including non-penguin species to better understand influenza virus ecology in Antarctic ecosystems.

The coexistence of endemic Low-Pathogenicity Avian Influenza (LPAI) lineages and the recently introduced clade 2.3.4.4b H5N1 raises concerns about potential genetic reassortment between low- and high-pathogenicity strains that could occur during coinfections. Experimental and field studies in other systems have demonstrated that influenza A viruses reassort efficiently when multiple strains coinfect the same host, particularly in wild birds and mammalian models [54,55,56]. Such scenarios underscore the importance of integrating active and serological surveillance, as reassortment risk depends on the temporal overlap of distinct influenza lineages circulating within host populations.

Taken together, these considerations highlight important limitations inherent to point-prevalence surveillance of avian influenza viruses in Antarctic ecosystems. Sampling restricted to a single breeding season and focused on apparently healthy adult birds represents a narrow temporal and demographic window, which may fail to capture short-lived circulation events or periods of increased viral shedding [57,58,59]. Viral shedding in wild birds is known to be temporally variable and strongly influenced by ecological factors such as seasonality, breeding dynamics, population density, and contact networks [60]. Consequently, while our findings provide an important snapshot of local viral circulation during the 2024/2025 austral summer, they underscore the need for sustained, multi-season, and multi-host surveillance approaches to more accurately characterize influenza A virus dynamics in Antarctica.

In this context, future surveillance efforts would benefit from the incorporation of longitudinal study designs that allow individual-level follow-up across seasons. Permanent marking approaches, such as banding of flying seabird species, could facilitate the re-identification of individuals and enable the assessment of temporal changes in infection status and antibody dynamics. While such approaches require specific permits, logistical capacity, and long-term monitoring frameworks under the Antarctic Treaty System, their implementation would substantially strengthen the interpretation of serological patterns and improve understanding of virus persistence, exposure history, and reinfection processes in Antarctic wildlife populations.

These limitations also apply to the detection of other respiratory viruses, including coronaviruses. The absence of molecular detection of coronaviruses in birds sampled at Lions Rump should be interpreted in the context of the still limited and uneven characterization of coronavirus circulation in Antarctic ecosystems. Importantly, deltacoronaviruses have already been detected in Antarctic seabirds, demonstrating that these viruses are present in the region [28,29,61]. However, detection has been inconsistent across sites, host species, and sampling periods. Available surveillance studies from the South Shetland Islands and other Antarctic regions have reported variable detection of avian coronaviruses, with some investigations yielding no detections in sampled birds and marine mammals, whereas others have documented the circulation and persistence of deltacoronaviruses within specific host populations [27,29,62,63].

At present, the limited number of available studies, together with modest and often penguin-dominated sample sizes and restricted geographic coverage, precludes robust conclusions regarding host range, prevalence, or spatial distribution of avian coronaviruses in Antarctica. Although coronavirus detections have so far been reported in a small number of species, including snowy sheathbills, kelp gulls, and gentoo penguins [27,28,29,63], this likely reflects sampling effort rather than true host restriction. As surveillance remains sparse, it is still too early to determine the extent to which coronavirus circulation is structured by host ecology, environmental conditions, or regional connectivity. Accordingly, the absence of detection in the present study should be interpreted as a consequence of limited sampling scope and timing, rather than as evidence of absence of coronaviruses in the broader Antarctic ecosystem.

From a One Health perspective, understanding the ecology of influenza A viruses and coronaviruses in Antarctica has implications that extend beyond wildlife health. Increasing human presence in the region through scientific research, logistics, and tourism creates opportunities for bidirectional pathogen exchange at the human, wildlife and environment interface, particularly in settings where close proximity to seabirds and marine mammals is unavoidable. Baseline knowledge of viral circulation in Antarctic wildlife is therefore essential to inform evidence-based biosecurity measures, reduce the risk of pathogen introduction or amplification, and protect both human health and fragile Antarctic ecosystems. In addition, Antarctica represents a unique natural laboratory for studying virus evolution, persistence, and dispersal under extreme environmental conditions, providing insights relevant to global understanding of zoonotic risk, viral genomics, and host–pathogen interactions. Integrating long-term, multi-host surveillance within a One Health framework is thus critical to support wildlife conservation, safeguard human activities, and anticipate future epidemiological scenarios in a rapidly changing polar environment.

## 5. Conclusions

This study provides important insights into avian viral dynamics in Antarctica in the context of the emergence of high-pathogenicity avian influenza (HPAI) H5N1 in the Antarctic Peninsula and South Shetland Islands. Following the initial incursion of HPAI into the region and its subsequent detection in multiple Antarctic species during preceding seasons, continued field-based surveillance has become essential to evaluate patterns of viral persistence, exposure, and potential re-introduction in Antarctic wildlife.

Although no active influenza A virus or coronavirus infections were detected by RT-PCR, the detection of antibodies in 11.3% of sampled birds demonstrates previous exposure to influenza A viruses, including subtypes H1, H5, H6, H9, and H11. These findings indicate that viral circulation has occurred despite the absence of detectable active infection during the expedition, reinforcing the importance of integrating molecular and serological approaches in Antarctic surveillance programs.

Importantly, the inclusion of multiple host species, encompassing not only penguins but also predators and scavengers such as skuas and sheathbills, enhances the ability of surveillance efforts to capture ecological pathways of viral maintenance, environmental contamination, and interspecies transmission. Protected breeding colonies within Antarctic Specially Protected Areas (ASPAs) therefore represent strategic sentinel sites for long-term monitoring of avian virus ecology and for assessing Antarctica’s role in global virus circulation. Sustained, multi-species surveillance across seasons will be critical to detect epidemiological changes, evaluate emerging risks, and support conservation and biosecurity strategies in one of the world’s most vulnerable and rapidly changing ecosystems.

## Figures and Tables

**Table 1:** Serological results of seabirds from the South Shetland Islands, Antarctica, showing reactivity to influenza A virus antibodies by enzyme-linked immunosorbent assay (ELISA) and hemagglutination inhibition (HI). Only ELISA-positive individuals are shown. For HI-reactive samples, subtype-specific HI titers are reported in parentheses. “Not reactive” indicates absence of hemagglutination inhibition at the screening dilution (1:16).

Bird ID	Species	ELISA	HI Subtypes Detected (HI Titers)
Av. 31	*Chionis albus*	Reactive	Not reactive
Av. 153	*Chionis albus*	Reactive	Not reactive
Av. 96	*Pygoscelis adeliae*	Reactive	Not reactive
Av. 108	*Pygoscelis adeliae*	Reactive	H6 (1:16), H11 (1:16)
Av. 133	*Pygoscelis adeliae*	Reactive	Not reactive
Av. 135	*Pygoscelis adeliae*	Reactive	Not reactive
Av. 180	*Pygoscelis adeliae*	Reactive	Not reactive
Av. 183	*Pygoscelis adeliae*	Reactive	Not reactive
Av. 52	*Pygoscelis antarcticus*	Reactive	H11 (1:16)
Av. 109	*Pygoscelis antarcticus*	Reactive	Not reactive
Av. 117	*Pygoscelis antarcticus*	Reactive	Not reactive
Av. 119	*Pygoscelis antarcticus*	Reactive	Not reactive
Av. 151	*Pygoscelis antarcticus*	Reactive	Not reactive
Av. 152	*Pygoscelis antarcticus*	Reactive	Not reactive
Av. 165	*Pygoscelis antarcticus*	Reactive	Not reactive
Av. 166	*Pygoscelis antarcticus*	Reactive	Not reactive
Av. 175	*Pygoscelis antarcticus*	Reactive	Not reactive
Av. 191	*Pygoscelis antarcticus*	Reactive	Not reactive
Av. 138	*Stercorarius antarcticus*	Reactive	H1 (≥1:32), H5 (1:16), H6 (1:16), H9 (1:16)
Av. 178	*Stercorarius antarcticus*	Reactive	Not reactive

## Data Availability

The original contributions presented in this study are included in the article. Further inquiries can be directed to the corresponding authors.

## Abbreviations

The following abbreviations are used in this manuscript:

IAV	Influenza A Virus
ASPAs	Antarctic Specially Protected Areas
CEUA	Animal Use Ethics Committee
CoVs	Coronaviruses
Ct	Cycle threshold
ELISA	Enzyme-Linked Immunosorbent Assay
HAU	Hemagglutinating Unit
HPAI	High-Pathogenicity Avian Influenza
HI	Hemagglutination Inhibition
LPAI	Low-Pathogenicity Avian Influenza
NP	Nucleoprotein
PROANTAR	Brazilian Antarctic Program
RT-PCR	Reverse Transcription Polymerase Chain Reaction
WOAH	World Organisation for Animal Health

## References

[1] Klaassen M., Wille M. (2023). The Plight and Role of Wild Birds in the Current Bird Flu Panzootic. Nat. Ecol. Evol..

[2] Ariyama N., Pardo-Roa C., Muñoz G., Aguayo C., Ávila C., Mathieu C., Almonacid L.I., Medina R.A., Brito B., Johow M. (2023). Highly Pathogenic Avian Influenza A (H5N1) Clade 2.3.4.4b Virus in Wild Birds, Chile. Emerg. Infect. Dis..

[3] Bruno A., Alfaro-Núñez A., De Mora D., Armas R., Olmedo M., Garcés J., Vaca M.S., De La Torre E., Jarrin D., Burbano L. (2023). Phylogenetic Analysis Reveals That the H5N1 Avian Influenza A Outbreak in Poultry in Ecuador in November 2022 Is Associated with the Highly Pathogenic Clade 2.3.4.4b. Int. J. Infect. Dis..

[4] Leguia M., Garcia-Glaessner A., Muñoz-Saavedra B., Juarez D., Barrera P., Calvo-Mac C., Jara J., Silva W., Ploog K., Amaro L. (2023). Highly Pathogenic Avian Influenza A (H5N1) in Marine Mammals and Seabirds in Peru. Nat. Commun..

[5] Banyard A.C., Bennison A., Byrne A.M.P., Reid S.M., Lynton-Jenkins J.G., Mollett B., De Silva D., Peers-Dent J., Finlayson K., Hall R. (2024). Detection and Spread of High Pathogenicity Avian Influenza Virus H5N1 in the Antarctic Region. Nat. Commun..

[6] Pardo-Roa C., Nelson M.I., Ariyama N., Aguayo C., Almonacid L.I., Gonzalez-Reiche A.S., Muñoz G., Ulloa M., Ávila C., Navarro C. (2025). Cross-Species and Mammal-to-Mammal Transmission of Clade 2.3.4.4b Highly Pathogenic Avian Influenza A/H5N1 with PB2 Adaptations. Nat. Commun..

[7] Uhart M.M., Vanstreels R.E.T., Nelson M.I., Olivera V., Campagna J., Zavattieri V., Lemey P., Campagna C., Falabella V., Rimondi A. (2024). Epidemiological Data of an Influenza A/H5N1 Outbreak in Elephant Seals in Argentina Indicates Mammal-to-Mammal Transmission. Nat. Commun..

[8] Dewar M., Wille M., Gamble A., Vanstreels R.E.T., Bouliner T., Smith A., Varsani A., Ratcliffe N., Black J., Lynnes A. (2023). The Risk of Highly Pathogenic Avian Influenza in the Southern Ocean: A Practical Guide for Operators and Scientists Interacting with Wildlife. Antarct. Sci..

[9] Kuiken T., Vanstreels R.E.T., Banyard A., Begeman L., Breed A.C., Dewar M., Fijn R., Serafini P.P., Uhart M., Wille M. (2025). Emergence, Spread, and Impact of High-pathogenicity Avian Influenza H5 in Wild Birds and Mammals of South America and Antarctica. Conserv. Biol..

[10] SCAR Sub-Antarctic and Antarctic Highly Pathogenic Avian Influenza H5N1 Monitoring Project. https://scar.org/library-data/avian-flu.

[11] Aguado B., Begeman L., Günther A., Iervolino M., Soto F., Vanstreels R.E.T., Reade A., Coerper A., Wallis B., Alcamí A. (2024). Searching for High Pathogenicity Avian Influenza Virus in Antarctica. Nat. Microbiol..

[12] Bennett-Laso B., Berazay B., Muñoz G., Ariyama N., Enciso N., Braun C., Krüger L., Barták M., González-Aravena M., Neira V. (2024). Confirmation of Highly Pathogenic Avian Influenza H5N1 in Skuas, Antarctica 2024. Front. Vet. Sci..

[13] McCulley M., Dewar M.L., Low Y.S., Wilson A., Jauregui R., Chernyavtseva A., O’Keefe J. (2025). High Pathogenicity Avian Influenza (HPAI) H5N1 Virus Detected in Brown Skua Using Portable Laboratory While at Sea in Antarctica. Microbiol. Resour. Announc..

[14] Morgan I.R., Westbury H.A. (1981). Virological Studies of *Adelie penguins* (*Pygoscelis adeliae*) in Antarctica. Avian Dis..

[15] Austin F.J., Webster R.G. (1993). EVIDENCE OF ORTHO- AND PARAMYXOVIRUSES IN FAUNA FROM ANTARCTICA. J. Wildl. Dis..

[16] Baumeister E., Leotta G., Pontoriero A., Campos A., Montalti D., Vigo G., Pecoraro M., Savy V. (2004). Serological Evidences of Influenza A Virus Infection in Antarctica Migratory Birds. Int. Congr. Ser..

[17] Miller G.D., Watts J.M., Shellam G.R. (2008). Viral Antibodies in South Polar Skuas around Davis Station, Antarctica. Antarct. Sci..

[18] Hurt A.C., Vijaykrishna D., Butler J., Baas C., Maurer-Stroh S., Silva-de-la-Fuente M.C., Medina-Vogel G., Olsen B., Kelso A., Barr I.G. (2014). Detection of Evolutionarily Distinct Avian Influenza A Viruses in Antarctica. mBio.

[19] Barriga G.P., Boric-Bargetto D., San Martin M.C., Neira V., Van Bakel H., Thompsom M., Tapia R., Toro-Ascuy D., Moreno L., Vasquez Y. (2016). Avian Influenza Virus H5 Strain with North American and Eurasian Lineage Genes in an *Antarctic penguin*. Emerg. Infect. Dis..

[20] De Seixas M.M.M., De Araújo J., Krauss S., Fabrizio T., Walker D., Ometto T., Matsumiya Thomazelli L., Vanstreels R.E.T., Hurtado R.F., Krüger L. (2022). H6N8 Avian Influenza Virus in Antarctic Seabirds Demonstrates Connectivity between South America and Antarctica. Transbounding Emerg. Dis..

[21] Hurt A.C., Su Y.C.F., Aban M., Peck H., Lau H., Baas C., Deng Y.-M., Spirason N., Ellström P., Hernandez J. (2016). Evidence for the Introduction, Reassortment, and Persistence of Diverse Influenza A Viruses in Antarctica. J. Virol..

[22] De Souza Petersen E., De Araujo J., Krüger L., Seixas M.M., Ometto T., Thomazelli L.M., Walker D., Durigon E.L., Petry M.V. (2017). First Detection of Avian Influenza Virus (H4N7) in Giant Petrel Monitored by Geolocators in the Antarctic Region. Mar. Biol..

[23] Ogrzewalska M., Couto Motta F., Resende P.C., Fumian T., Fonseca Da Mendonça A.C., Appolinario Reis L., Lima Brandao M., Chame M., Arantes Gomes I.L., Mendonca Siqueira M. (2022). Influenza A (H11N2) Virus Detection in Fecal Samples from Adélie (*Pygoscelis adeliae*) and Chinstrap (*Pygoscelis antarcticus*) Penguins, Penguin Island, Antarctica. Microbiol. Spectr..

[24] Ohlopkova O.V., Goncharov A.E., Aslanov B.I., Fadeev A.V., Davidyuk Y.N., Moshkin A.D., Stolbunova K.A., Stepanyuk M.A., Sobolev I.A., Tyumentseva M.A. (2024). First Detection of Influenza A Virus Subtypes H1N1 and H3N8 in the Antarctic Region: King George Island, 2023. Probl. Virol..

[25] Miłek J., Blicharz-Domańska K. (2018). Coronaviruses in Avian Species—Review with Focus on Epidemiology and Diagnosis in Wild Birds. J. Vet. Res..

[26] Wille M., Shi M., Klaassen M., Hurt A.C., Holmes E.C. (2019). Virome Heterogeneity and Connectivity in Waterfowl and Shorebird Communities. ISME J..

[27] Wille M., Holmes E.C. (2020). Wild Birds as Reservoirs for Diverse and Abundant Gamma- and Deltacoronaviruses. FEMS Microbiol. Rev..

[28] Zamora G., Aguilar Pierlé S., Loncopan J., Araos L., Verdugo F., Rojas-Fuentes C., Krüger L., Gaggero A., Barriga G.P. (2023). Scavengers as Prospective Sentinels of Viral Diversity: The Snowy Sheathbill Virome as a Potential Tool for Monitoring Virus Circulation, Lessons from Two Antarctic Expeditions. Microbiol. Spectr..

[29] Gomes F., Freitas Da Silva A., Prado T., Resende P.C., Corrêa Da Silva Junior L., Degrave W., Magalhães M., Vivioni A., Mariz Dias Y.J., Siqueira M. (2025). Long-Term Maintenance of a *Deltacoronavirus* Infecting Multiple Bird Species in Antarctica. Microbiol. Spectr..

[30] Rahman M.M., Talukder A., Chowdhury M.M.H., Talukder R., Akter R. (2021). Coronaviruses in Wild Birds—A Potential and Suitable Vector for Global Distribution. Vet. Med. Sci..

[31] Antarctic Treaty Consultative Meeting (2024). Management Plan for Antarctic Specially Protected Area No. 151 (Lions Rump, King George Island, South Shetland Islands).

[32] Matsuoka K., Skoglund A., Roth G., de Pomereu J., Griffiths H., Headland R., Herried B., Katsumata K., Le Brocq A., Licht K. (2021). Quantarctica, an integrated mapping environment for Antarctica, the Southern Ocean, and sub-Antarctic islands. Environ. Model. Softw..

[33] Shu B., Wu K.-H., Emery S., Villanueva J., Johnson R., Guthrie E., Berman L., Warnes C., Barnes N., Klimov A. (2011). Design and Performance of the CDC Real-Time Reverse Transcriptase PCR Swine Flu Panel for Detection of 2009 A (H1N1) Pandemic Influenza Virus. J. Clin. Microbiol..

[34] Chu D.K.W., Leung C.Y.H., Gilbert M., Joyner P.H., Ng E.M., Tse T.M., Guan Y., Peiris J.S.M., Poon L.L.M. (2011). Avian Coronavirus in Wild Aquatic Birds. J. Virol..

[35] (2024). WOAH Codes and Manuals.

[36] Bennison A., Adlard S., Banyard A.C., Blockley F., Blyth M., Browne E., Day G., Dunn M.J., Falchieri M., Fitzcharles E. (2024). A Case Study of Highly Pathogenic Avian Influenza (HPAI) H5N1 at Bird Island, South Georgia: The First Documented Outbreak in the Subantarctic Region. Bird. Study.

[37] Ogrzewalska M., Pereira E.C., Vanstreels R.E.T., Campista E., Correa Junior L., Macedo L., Appolinario L.R., Brandão M.L., Vilela R., Degrave W. (2024). High Pathogenicity Avian Influenza Virus (HPAIV) H5N1 Clade 2.3.4.4b Recovered from a Kelp Gull (*Larus dominicanus*) in the South Shetland Islands, Antarctica. bioRxiv.

[38] León F., Le Bohec C., Pizarro E.J., Baille L., Cristofari R., Houstin A., Zitterbart D.P., Ulloa-Contreras C., Barriga G., Poulin E. (2025). Tracking HPAIV H5 through a Geographic Survey of Antarctic Seabird Populations. Sci. Rep..

[39] Xu R., Gao M., Zhang N., Wei Z., Wang Z., Zhang L., Liu Y., Zheng Z., Chen L., Ding H. (2025). Transcontinental Spread of HPAI H5N1 from South America to Antarctica via Avian Vectors. Viruses.

[40] Ogrzewalska M., Vanstreels R.T., Pereira E., Campinas E., Junior L.C., Melo J.O., Macedo L., Appolinario L., Arantes I., Brandao M.L. (2025). Genomic Analysis of High Pathogenicity Avian Influenza Viruses from Antarctica Reveals Multiple Introductions from South America. Prepr. Res. Sq..

[41] CDC Manual for the Laboratory Diagnosis and Virological Surveillance of Influenza. https://stacks.cdc.gov.

[42] WOAH Manual of Diagnostic Tests and Vaccines for Terrestrial Animals 2021—6th Edition. https://www.woah.org/fileadmin/Home/eng/Health_standards/tahm/A_summry.htm.

[43] Spackman E., Senne D.A., Bulaga L.L., Myers T.J., Perdue M.L., Garber L.P., Lohman K., Daum L.T., Suarez D.L. (2003). Development of Real-Time RT-PCR for the Detection of Avian Influenza Virus. Avian Dis..

[44] Wallensten A., Munster V.J., Osterhaus A.D.M.E., Waldenström J., Bonnedahl J., Broman T., Fouchier R.A.M., Olsen B. (2006). Mounting Evidence for the Presence of Influenza A Virus in the Avifauna of the Antarctic Region. Antart. Sci..

[45] Abad F.X., Busquets N., Sanchez A., Ryan P.G., Majó N., Gonzalez-Solís J. (2013). Serological and Virological Surveys of the Influenza A Viruses in Antarctic and Sub-Antarctic Penguins. Antarct. Sci..

[46] Morgan I.R., Westbury H.A., Ferris J.M., Burton H.R., Johnstone G.W., Bayly I.A.E. (1988). Studies of Viruses in Penguins in the Vestfold Hills. Biology of the Vestfold Hills, Antarctica.

[47] Trivelpiece W.Z., Bengtson J.L., Trivelpiece S.G., Volkman N.J. (1986). Foraging Behavior of Gentoo and Chinstrap Penguins as Determined by New Radiotelemetry Techniques. Auk.

[48] Wienecke B.C., Lawless R., Rodary D., Bost C.-A., Thomson R., Pauly T., Robertson G., Kerry K.R., LeMaho Y. (2000). Adélie Penguin Foraging Behaviour and Krill Abundance along the Wilkes and Adélie Land Coasts, Antarctica. Deep. Sea Res. Part. II Top. Stud. Oceanogr..

[49] Michelot C., Kato A., Raclot T., Ropert-Coudert Y. (2021). Adélie Penguins Foraging Consistency and Site Fidelity Are Conditioned by Breeding Status and Environmental Conditions. PLoS ONE.

[50] Reinhardt K., Hahn S., Peter H.-U., Wemhoff H. (2000). A Review Of The Diets Of Southern Hemisphere Skuas. Mar. Ornithol..

[51] Golubev S. (2024). Diet and Feeding Behavior of the South Polar Skuas Stercorarius Maccormicki in the Haswell Islands, East Antarctica. Birds.

[52] BirdLife International BirdLife DataZone. https://datazone.birdlife.org/search.

[53] Camphuysen C.J., Gear S.C., Furness R.W. (2022). Avian Influenza Leads to Mass Mortality of Adult Great Skuas in Foula in Summer 2022. Scott. Birds.

[54] Gong X., Hu M., Chen W., Yang H., Wang B., Yue J., Jin Y., Liang L., Ren H. (2021). Reassortment Network of Influenza A Virus. Front. Microbiol..

[55] Ganti K., Bagga A., Carnaccini S., Ferreri L.M., Geiger G., Joaquin Caceres C., Seibert B., Li Y., Wang L., Kwon T. (2022). Influenza A Virus Reassortment in Mammals Gives Rise to Genetically Distinct Within-Host Subpopulations. Nat. Commun..

[56] Taylor K.Y., Agu I., José I., Mäntynen S., Campbell A.J., Mattson C., Chou T.-W., Zhou B., Gresham D., Ghedin E. (2023). Influenza A Virus Reassortment Is Strain Dependent. PLoS Pathog..

[57] Hoye B.J., Munster V.J., Nishiura H., Klaassen M., Fouchier R.A.M. (2010). Surveillance of Wild Birds for Avian Influenza Virus. Emerg. Infect. Dis..

[58] Giacinti J.A., Robinson S.J., Sharp C.M., Provencher J.F., Pearl D.L., Stevens B., Nituch L., Brook R.W., Jardine C.M. (2024). Assessing Avian Influenza Surveillance Intensity in Wild Birds Using a One Health Lens. One Health.

[59] Martelli L., Fornasiero D., Martínez-Lanfranco J.A., Spada A., Scarton F., Scolamacchia F., Manca G., Mulatti P. (2025). Exploring the Role of Wild Bird Species in the Transmission of Avian Influenza to Poultry. Transbound. Emerg. Dis..

[60] Blagodatski A., Trutneva K., Glazova O., Mityaeva O., Shevkova L., Kegeles E., Onyanov N., Fede K., Maznina A., Khavina E. (2021). Avian Influenza in Wild Birds and Poultry: Dissemination Pathways, Monitoring Methods, and Virus Ecology. Pathogens.

[61] Wille M., Harvey E., Shi M., Gonzalez-Acuña D., Holmes E.C., Hurt A.C. (2020). Sustained RNA Virome Diversity in *Antarctic penguins* and Their Ticks. ISME J..

[62] Gomes F., Prado T., Degrave W., Moreira L., Magalhães M., Magdinier H., Vilela R., Siqueira M., Brandão M., Ogrzewalska M. (2023). Active Surveillance for Influenza Virus and Coronavirus Infection in Antarctic Birds and Mammals in Environmental Fecal Samples, South Shetland Islands. An. Acad. Bras. Ciênc..

[63] Gomes F., da Silva A.F., Prado T., Resende P.C., Silva L.C.D., Degrave W., Magalhães M., Vivioni A., Dias Y.J.M., Siqueira M. (2024). Detection and Full Genome Sequencing of a Deltacoronavirus and Other Bird Associated Viruses from Feces of the Kelp Gull (*Larus dominicanus*) Sampled at the South Shetland Islands Antarctica. bioRxiv.

